# Evaluation of Interactive Voice Response (IVR) and postal survey in follow-up of children and adolescents discharged from psychiatric outpatient treatment: a randomized controlled trial

**DOI:** 10.1186/2193-1801-3-77

**Published:** 2014-02-08

**Authors:** Claes Andersson, Susanne Danielsson, Gunilla Silfverberg-Dymling, Gunnel Löndahl, Björn Axel Johansson

**Affiliations:** Health and Society, Department of Criminology, Malmö University, SE-205 06 Malmö, Sweden; Division of Psychiatry, Department of Clinical Sciences, Lund University, Lund, Sweden; Psychiatry Skåne, Department of Child & Adolescent Psychiatry, Skåne University Hospital, Malmö, Sweden; Division of Child & Adolescent Psychiatry, Department of Clinical Sciences, Lund University, Lund, Sweden; Clinical Health Promotion Centre, Department of Health Sciences, Lund University, Lund, Sweden

**Keywords:** Child and adolescent psychiatry, Evaluation, Interactive voice response (IVR), Postal survey, Brief child and family phone interview (BCFPI)

## Abstract

Systematic evaluation of child and adolescent psychiatric outpatient treatment is important but time-consuming. The aim of this paper was to study whether Interactive Voice Response (IVR) is a more effective method than a questionnaire sent by post when following up outpatient treatment in child and adolescent psychiatry. Eighty patients were recruited from a child and adolescent psychiatric outpatient unit in Sweden. One parent of each of the patients was randomized to complete the BCFPI follow-up form, using either IVR (n = 40) or postal survey (n = 40) one month after discharge. The response rate for complete answers was 65% in the IVR group and 38% in the postal survey group (p = 0.014). There was less need for reminders in the IVR group (p = 0.000). IVR is a promising and cost-effective method for evaluating evidence-based treatment in child and adolescent psychiatric care.

## Introduction

Approximately 15% of all children and adolescents have a mental disorder that requires treatment (Blakemore 2008). Common psychiatric syndromes are anxiety, depressive disorders and hyperactivity (Ries Merkangas et al. 2009), causing suffering for the young person in question as well as for their relatives and peers (Weisz et al. 2011). Evidence-based treatment options are available for children and adolescents with psychiatric disorders (The Cochrane Collaboration 2013). The concept of evidence-based psychiatry does not only include the treatment: it also emphasises the importance of continuous and systematic evaluation of the treatment options to decide whether improvements are needed (Akobeng 2005). In child and adolescent psychiatry there are validated instruments to evaluate treatment (Dulcan 2010). One example is the Brief Child and Family Phone Interview (BCFPI), which is a structured survey for clinical intake evaluation, treatment planning and follow-up assessment (Cunningham et al. 2009; Boyle et al. 2009). The interview involves parents, teachers, and adolescents. BCFPI is primarily designed as a phone interview performed by a clinical interviewer with formal training in children’s mental health. According to the manual, the assessment takes approximately 30 minutes to complete (Cunningham et al. 2006), but our experience is that it takes longer, sometimes up to 60 minutes. The overall burden, not only on the departments but also on the individual clinicians, increases the risk that treatment evaluations will not be prioritized. To increase the probability of the evaluation being carried out, various kinds of automated technologies can be used to perform clinical evaluations. Interactive Voice Response (IVR) is an automated telephone system where a central computer is programmed to administer questionnaires; respondents answer pre-recorded questions by pressing a number on the keypad. All answers are stored directly in the central computer. In a limited number of studies, IVR has been used to follow up adolescents in psychiatric inpatient and outpatient care (Johansson et al. 2013), but there are no previous studies comparing IVR and postal surveys when following up outpatient treatment in child and adolescent psychiatry.

### Aim

The primary aim was to investigate whether IVR is a more effective method than postal surveys when collecting data for use in evaluating outpatient treatment in child and adolescent psychiatry. A secondary aim was to evaluate whether IVR requires less reminders compared to postal surveys.

## Methods

About 250 000 children and adolescents live in the catchment area of the Department of Child and Adolescent Psychiatry, Malmö, Sweden. As part of ongoing developmental work, the department used the BCFPI parent interview as intake screening and as a follow-up instrument one month after discharge from treatment. In the current study a total of 80 parents to consecutive patients who had completed outpatient treatment during March-September 2008 were randomized to a BCFPI parent follow-up assessment with either IVR or a postal survey. For inclusion in the study, parents had to understand written and spoken Swedish. Those randomized to the IVR group (n = 40) received a letter informing them about the evaluation and giving a specific date and time for the automated phone interview. The IVR system was programmed to make eight attempts during the evening if the parent did not respond. If the call was answered, a short introduction was given including a confirmation that the person answering was the one who completed the BCFPI intake interview, and then the BCFPI survey took place. Those randomized to the postal survey (n = 40) received a similar letter, but also including the BCFPI questionnaire. The parent was asked to complete the form and return it to the department using a pre-paid envelope. Regardless of randomization group, one reminder with the same procedure was sent by the department staff if the parent did not respond.

### Statistics

Fisher’s exact test was used to compare frequencies of response rates and reminders. Descriptive statistics are presented as mean (SD) for continuous variables and as counts for categorical data. All tests were two-tailed. A p-value of <0.05 was regarded significant (Altman 1990). SPSS version 20 was used for the statistical calculations.

## Results and discussion

IVR proved to be more effective than postal surveys when using the BCFPI parent survey to follow up children and adolescents who had completed psychiatric outpatient treatment. In the IVR group, 26 parents responded to the automated phone interview, while 19 responses were received from the postal survey group (p = 0.115). There was a higher proportion of complete answers in the IVR group (26/40, 65%), compared to the postal survey group (15/40, 38%) (p = 0.014). The excluded surveys, i.e. insufficiently-completed BCFPI forms, could not be used to evaluate treatment. In the IVR group, 17 parents received a reminder, compared to 36 parents in the postal survey group (p = 0.000), i.e. the department spent less time on sending reminders to participants in the IVR group. The mean (SD) time for the IVR respondents was 10:57 (1:40) minutes, compared with the 30–60 minutes needed for a phone interview.

There are several possible explanations for the higher rate of complete answers and for the reduced need for reminders in the IVR group. IVR requires an immediate answer, while the postal survey may easily be postponed or forgotten. Automated telephony is probably more challenging and offers less opportunity to omit certain items. The IVR responses are delivered instantly, while subjects in the other group have to put in additional effort to physically complete and then post the questionnaire. The reason for the reduced time consumption for the respondents compared to that estimated for a clinical phone interview is probably that initial 'small talk’ and comments between the answers in a person-to-person phone interview are omitted in the IVR procedure. The IVR superiority may increase the probability that treatment is actually evaluated, thereby releasing more time for clinical work for the trained clinicians.

There are other advantages of using the IVR technology. The automated phone survey can be implemented without pre-training of the respondent, and data is stored directly in the central computer, allowing immediate analysis and feedback to clinicians.

IVR reduces the need for clinicians to engage in administrative tasks and check that data is collected, although data ultimately has to be interpreted and managed by clinicians. IVR uses validated instruments but in other areas, such as the diagnostic process, clinicians are still needed. When using validated instruments, it is important that questions are asked according to the manual, i.e. that data is not biased by the interviewer. IVR manages closed-response alternatives, including multiple-response options, e.g. patient medication, but can also administer qualitative, open questions by using recordings. Like postal surveys, IVR has limited possibilities for additional follow-up questions as each question is pre-programmed, but both methods allow additional comments.

### Strengths

To our knowledge this is the first randomized study evaluating children and adolescents discharged from psychiatric outpatient treatment with IVR carrying a validated screening instrument.

### Limitations

A limitation is that background data including diagnoses and treatment regimes, which could possibly explain some group differences, was not collected. Data on the amount of time needed for completing the paper survey was not obtained. A third study arm involving a person-to-person telephone BCFPI follow-up survey could have better answered the question of whether IVR follow-up is less time consuming. The study did not examine whether BCFPI follow-up with IVR and the paper survey have the same quality as an interview performed by a trained clinician.

### Future implications

The IVR technology is easily implemented, and may be used to further evaluate and develop evidence-based treatment models in child and adolescent psychiatry. The IVR system could probably provide targeted feedback to the parent based on the BCFPI follow-up result, e.g. congratulate a positive or stable result, or recommend support for those with a negative outcome.

## Conclusion

IVR is a promising and cost-effective method offering high response rates and little burden on clinical staff and respondents. The technology is not personnel-dependent and could easily be implemented in child and adolescent psychiatric care to evaluate evidence-based treatment.

### Ethics

According to Swedish legalisation, ethical approval is not required for developmental work. The study fully complies with the guidelines of the Committee of Ethics in Sweden. No compensation was offered to the participants.
